# Cognitive and Motor Outcomes of Children With Prenatal Opioid Exposure: A Systematic Review and Meta-analysis

**DOI:** 10.1001/jamanetworkopen.2019.7025

**Published:** 2019-07-12

**Authors:** Su Lynn Yeoh, John Eastwood, Ian M. Wright, Rachael Morton, Edward Melhuish, Meredith Ward, Ju Lee Oei

**Affiliations:** 1 Medical student, University of New South Wales, Sydney, New South Wales, Australia; 2 Sydney Local Health District, Croydon, New South Wales, Australia; 3 Ingham Institute of Applied Medical Research, Liverpool, New South Wales, Australia; 4 Sydney Medical School, Faculty of Medicine and Health, Sydney University, Sydney, New South Wales, Australia; 5 Sydney Institute for Women, Children and Their Families, Camperdown, Sydney, New South Wales, Australia; 6 Early Start Research Institute, University of Wollongong, Wollongong, New South Wales, Australia; 7 University of Queensland Centre for Clinical Research, Herston, Queensland, Australia; 8 NHMRC Clinical Trials Centre, Faculty of Medicine and Health, University of Sydney, Camperdown, New South Wales, Australia; 9 Department of Education, University of Oxford, Oxford, United Kingdom; 10 Birkbeck, University of London, London, United Kingdom; 11 Department of Newborn Care, Royal Hospital for Women, Sydney, Australia

## Abstract

**Importance:**

Prenatal opioid exposure (POE) is one of the fastest-growing global health problems, but its association with long-term neurologic and physical development remains unknown.

**Objective:**

To assess the association between POE and cognitive and motor development in children from age 6 months to 18 years.

**Data Sources:**

Key search terms included *prenatal opioid exposure*, *neonatal abstinence syndrome*, and *neurocognitive development*. Studies were searched using PubMed and Embase, with no publication date restriction, through August 20, 2018.

**Study Selection:**

Only published cohort studies comparing the results of age-appropriate standardized cognitive and/or motor tests between children with any POE (aged 0-18 years) with drug-free controls were included. Data that were not convertible to means and SDs were excluded.

**Data Extraction and Synthesis:**

This study was conducted according to Preferred Reporting Items for Systematic Reviews and Meta-analyses (PRISMA) and Meta-analysis of Observational Studies in Epidemiology (MOOSE) guidelines. Data were pooled using a random-effects model.

**Main Outcomes and Measures:**

Standardized mean difference of cognitive and motor tests between POE and nonexposed children.

**Results:**

Twenty-six peer-reviewed cohort studies were included. Cognitive outcomes were compared for a total of 1455 children with POE and 2982 nonexposed children across 3 age groups (mean [SE] age at cognitive testing was 13 [1.58] months for the toddler group; 4.5 [0.38] years for the preschool group; and 13 [2.36] years for the school-aged group). Motor outcomes were compared for 688 children with POE and 1500 nonexposed children up to age 6 years (mean [SD] age at motor testing, 2 [0.45] years). Standardized mean difference was lower in cognitive tests for children with POE at 0 to 2 years (*d* = −0.52; 95% CI, −0.74 to −0.31; *P* < .001) and 3 to 6 years (*d* = −0.38; 95% CI, −0.69 to −0.07; *P* < .001); the difference was not significant for those aged 7 to 18 years (*d* = −0.44; 95% CI, −1.16 to 0.28; *P* = .23). Motor scores were lower in children with POE (*d* = 0.49; 95% CI, 0.23-0.74; *P* < .001).

**Conclusions and Relevance:**

Prenatal opioid exposure appeared to be negatively associated with neurocognitive and physical development from age 6 months, and this association persisted until adolescence. The cause and association of this with POE or other factors (eg, withdrawal treatment) are uncertain but suggest that POE necessitates long-term support and intervention.

## Key Points

**Question:**

Is prenatal opioid exposure associated with differences in childhood cognitive and motor development?

**Findings:**

In this systematic review and meta-analysis of 26 studies including 1455 children exposed to prenatal opioids compared with unexposed children, prenatal opioid exposure was associated with lower cognitive scores. The largest difference was seen between ages 6 months and 6 years.

**Meaning:**

The negative consequences of prenatal opioid exposure on neurocognitive and physical development appear to be present from 6 months and persist beyond school age.

## Teaser

This systematic review and meta-analysis examines the cognitive and motor development of infants and children exposed to opioids prenatally.

## Introduction

Prenatal opioid exposure (POE) is a fast-growing health problem, with at least 1 in 5 pregnant women in high-income countries known to have used some form of opioid during pregnancy.^1^ This incidence has been reported to be associated with increases in the risk of perinatal problems, including neonatal abstinence syndrome (NAS), prematurity, and low birth weight.^2^ Neonatal abstinence syndrome affects 75% to 90% of all infants with POE^1^ and is considered a major global public health issue. The number of babies affected by NAS has increased by more than 400% in the past 2 decades,^3^ resulting in consumption of health care and social resources. Public expenditure on hospital care for newborns with NAS in the United States alone exceeds $1 billion US dollars per year.^3^

The outcomes of infants with POE are therefore relevant, especially in regard to neurodevelopment. In animal studies, opioids impair neuronal development, differentiation, growth, and survival,^4,5^ as well as neurotransmitter homeostasis.^6,7^ Changes in brain volume and function are evident even after short-term opioid use in adult humans.^8^ Prenatal opioid exposure is also associated with a higher risk of exposure to adverse social, environmental, and familial disadvantages that may impede optimal neurodevelopment.^9^ For example, opioid-using mothers often have poorer educational attainment,^10^ an increased risk of psychiatric comorbidity,^11^ and poorer physical health^12^ that, together with other problems (eg, poverty, inadequate nutrition, and social chaos), may impair their ability to nurture their children.

There are minimal data on long-term outcomes of children with POE. Most children with POE are healthy and have no other medical issues, making the expense for long-term follow-up difficult to justify.^13^ Families affected by POE may also be mobile. In Australia, more than 50% of children of mothers in the methadone program are placed in foster care by age 5 and are subjected to various home placements and name changes,^14^ making long-term tracking difficult.

Nevertheless, there is increasing evidence that neurodevelopmental surveillance and intervention for children with POE should be as important as follow-up for children with other problems (eg, prematurity). Opioids cross the placental and milk barriers and are easily detectable in newborn and fetal products.^15^ The exact association between opioids and neurogenesis and function is unclear, but opioids have been shown to induce apoptosis of human brain cell cultures in vitro^5^ and impair synaptosomal uptake of neurotransmitters, such as dopamine and norepinephrine, in mice.^7^ In human studies, children with a history of POE have smaller head circumferences^16^ and lower brain volumes, especially of the basal ganglia and cerebellum,^17^ than other children, and these changes persist to adolescence.^18^ The association with function is unclear, but in the general population, smaller brain volumes are reported as being associated with lower intelligence and cognitive skills.^19^

Individual neurodevelopmental tests are robust indicators of child functioning. They serve to inform on the developmental needs of the child so that intervention therapies can be provided to decrease the risk of future functional problems. However, these tests are time consuming and difficult to conduct, especially with a mobile and chaotic population. Currently available neurodevelopmental data for children with POE arise from small, heterogeneous studies that, individually, are inadequately powered to inform on the needs of this group of children.

We therefore conducted a systematic review and meta-analysis of cohort studies to determine whether association exists between POE and neurodevelopmental outcomes in children aged 0 to 18 years. We hypothesized that POE will be negatively associated with long-term cognitive and motor outcomes and that this association will be apparent before the child enters school.

## Methods

This systematic review and meta-analysis was conducted and reported using the guidelines for Meta-analysis of Observational Studies in Epidemiology (MOOSE)^20^ and the Preferred Reporting Items for Systematic Reviews and Meta-analyses (PRISMA) reporting guideline.^21^

### Eligibility Criteria

**Figure 1.**
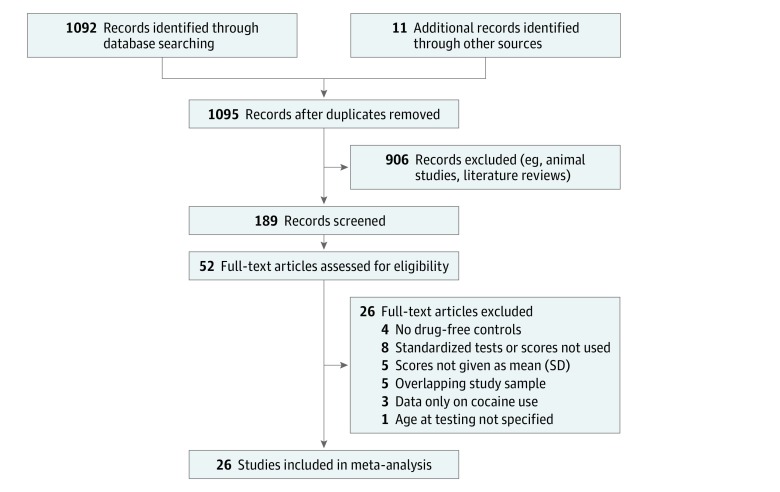
PRISMA Flow Diagram of Search

Only published cohort studies that compared outcomes of children with POE (aged 0-18 years) with drug-free controls were included. Included studies measured cognitive and/or motor development using age-appropriate, standardized tests and reported results as a mean and SD. Studies without enough data necessary to derive the mean and SD were excluded. All types of opioids were included, such as heroin, methadone, and buprenorphine, as well as known polysubstances (ie, >1 class of drug). Studies were excluded if they did not include human participants, were literature reviews, and did not have drug-free controls as comparators (Figure 1). To our knowledge, there have been no randomized clinical trials of POE vs no exposure.

### Electronic Search Method and Study Selection

Electronic databases (PubMed and Embase) were searched comprehensively by 2 of us independently (S.L.Y., J.L.O.). Hand searching was also conducted for references of included studies and those of relevant reviews. Backward searching looking for other articles by the same authors was also used, especially for longitudinal cohort studies. A strategy using the search terms *prenatal exposure*, *opioid*, *methadone*, *heroin*, *neonatal abstinence syndrome*, *cognition*, *school*, *academic achievement*, *intelligence*, and *neurodevelopment* was conducted with no publication date restriction. Articles had to be published in English and as the complete study. The initial search began on June 12, 2018, and continued concurrently with data extraction until August 10, 2018. Three of us (S.L.Y, R.M., and J.L.O.) assessed eligibility by title and abstract screening, and any discrepancies were discussed among all authors with a full-text article review. Study authors were not contacted for further information owing to the protracted amount of time from when some of the studies were conducted (>20 years).

### Data Extraction

Data extracted from each eligible study included type of exposure to opioids and other drugs, place of birth, rate of NAS, rate of out-of-home placement, age, and types of neurodevelopmental tests used and their outcomes. For longitudinal studies that assessed children several times over years and if results were published in 1 or multiple articles, only 1 result was selected for each age subgroup. For cognitive outcomes, selection was based first on the largest sample size followed by the age closest to the mean age for that subgroup. For motor outcomes, selection was based on the largest sample size followed by the most recent test.^22^ The subgroups were infancy (≤24 months), preschool age (3-6 years), and school age (7-18 years).

### Statistical Analysis

The main outcome measures were standardized mean differences (SMDs) and 95% CIs, calculated from the means and SDs of neurodevelopmental tests for POE and unexposed children. Standard meta-analytic procedures were conducted with the Cochrane Collaboration Review Manager Software (RevMan, version 5.3) and Meta-Analyst.^23^ Publication bias and funnel plots were assessed and generated using Meta-Essentials. Because the studies used different assessment tools, a random effects model^24^ was used to calculate SMD, which was used as effect size per Cohen *d* (0.3-0.4, small; 0.5-0.8, moderate; >0.8, large effect).^25^ Publication bias was assessed visually by looking for asymmetry in funnel plots and formally with the Egger test. The Egger test is a linear regression test that examines the association between effect size and SE and is used together with a funnel plot because visual assessment can be subjective.^26^ Study heterogeneity was assessed using *I*^2^ analysis. Heterogeneity was considered significant if the *I*^2^ value was greater than 50%.^27^ The quality of the included articles was assessed using the Newcastle-Ottawa Scale,^28^ which was originally a 9-point scale system, but one that we adapted to 7 points. The criteria of demonstration that outcome of interest was not present at the start of the study and follow-up was long enough for outcomes to occur were excluded as they were not applicable to the outcome of neurocognitive development. A score of 5 was the threshold for a study to be considered high quality. Sensitivity analyses using only high-quality studies were conducted to determine whether the effect size changed.

### Additional Analyses

Subgroup analyses based on opioid type, test used, and whether the study controlled for socioeconomic status were performed to examine whether the status contributed to study heterogeneity. Details are provided in Table 1 and Table 2.^27^ Post hoc random-effects metaregression analysis was performed to identify the association of clinical factors, such as rates of NAS, sex, and foster care, with differences in effect size. Additional analyses were performed only on the 6- to 24-month and 3- to 6-year age groups because of the adequate number of studies with sufficiently large samples (n > 10).

**Table 1.** Cognitive Subgroup Analyses

Variable	Subgroup	Ages 6-24 mo				Ages 3-6 y			
		No. of Studies	SMD (95% CI)	*I*^2^, %	*P* Value for Heterogeneity	No. of Studies	SMD (95% CI)	*I*^2^, %	*P* Value for Heterogeneity
Overall	NA	13	−0.52 (−0.74 to −0.31)	71	<.001	13	−0.38 (−0.69 to −0.0.7)	86	<.001
Main opioid used	Methadone	10	−0.61 (−0.88 to −0.33)	72	<.001	7	−0.52 (−0.78 to −0.27)	55	.04
	Heroin	1	−0.54 (−0.89 to −0.18)	NA	NA	4	−0.41 (−0.64 to −0.17)	0	.79
	Unspecified	2	−0.22 (−0.43 to −0.013)	0	.53	2	0.24 (−0.66 to 1.13)	93	<.001
Controlled for SES	Yes	8	−0.47 (−0.76 to −0.17)	71	.001	8	−0.20 (−0.59 to 0.19)	88	<.001
	No	5	−0.62 (−0.91 to −0.33)	62	.03	5	−0.70 (−0.91 to −0.48)	86	<.001
Test used	BSID	8	−0.40 (−0.60 to −0.20)	35	.15	0	NA	NA	NA
	BSID-II	3	−0.57 (−1.00 to −0.14)	83	.003	0	NA	NA	NA
	BSID-III	1	−2.25 (−3.06 to −1.44)	NA	NA	0	NA	NA	NA
	Griffiths Mental Development Scales	1	−0.50 (−0.99 to −0.010)	NA	NA	0	NA	NA	NA
	Stanford-Binet Intelligence Scales	0	NA	NA	NA	4	−0.38 (−0.55 to −0.20)	0	.70
	MSCA	0	NA	NA	NA	5	−0.43 (−0.77 to −0.08)	60	.04
	BSID-II	0	NA	NA	NA	1	0.68 (0.45 to 0.91)	NA	NA
	MPMST	0	NA	NA	NA	1	−0.77 (−1.32 to −0.22)	NA	NA
	SON	0	NA	NA	NA	1	−0.96 (−1.53 to −0.40)	NA	NA
	WPPSI-III	0	NA	NA	NA	1	−0.57 (−1.39 to 0.25)	NA	NA

Abbreviations: BSID, Bayley Scales of Infant and Toddler Development; BSID-II, Bayley Scales of Infant and Toddler Development–Second Edition; BSID-III, Bayley Scales of Infant and Toddler Development–Third Edition; MPMST, Merill-Palmer Scale of Mental Tests; MSCA, McCarthy Scales of Children’s Abilities; NA, not applicable; SES, socioeconomic status; SMD, standardized mean difference; SON, Snijders-Oomen Nonverbal Intelligence scale; WPPSI-III, Wechsler Preschool and Primary Scale of Intelligence, third edition.

**Table 2.** Motor Subgroup Analyses

Variable	Subgroup	Ages 6-24 mo			
		No. of Studies	SMD (95% CI)	*I*^2^, %	*P* Value for Heterogeneity
Overall	NA	14	−0.49 (−0.74 to −0.23)	80	<.001
Main opioid used	Methadone	9	−0.66 (−1.05 to −0.28)	84	<.001
	Heroin	3	−0.47 (−0.74 to −0.20)	22	.28
	Unspecified	2	−0.03 (−0.26 to 0.19)	57	.30
Controlled for SES	Yes	8	−0.65 (−1.05 to −0.24)	86	<.001
	No	6	−0.35 (−0.68 to −0.01)	73	<.001
Test used	BSID	7	−0.33 (−0.54 to −0.12)	37	.14
	BSID-II	2	−0.39 (−1.01 to 0.22)	89	.002
	MSCA	2	−0.30 (−1.18 to 0.58)	83	.01
	BSID-III	1	−3.50 (−4.5 to −2.54)	NA	NA
	Griffiths Mental Development Scales	1	−0.67 (−1.16 to −0.17)	NA	NA
	Purdue Pegboard Test	1	−0.34 (−0.74 to 0.063)	NA	NA

Abbreviations: BSID, Bayley Scales of Infant and Toddler Development; BSID-II, Bayley Scales of Infant and Toddler Development–Second Edition; BSID-III, Bayley Scales of Infant and Toddler Development–Third Edition; MSCA, McCarthy Scales of Children’s Abilities; NA, not applicable; SES, socioeconomic status; SMD, standardized mean difference.

## Results

### Study Selection

There were 26 studies eligible for inclusion in the meta-analysis. The database search identified 1103 citations. After removal of duplicates, 1095 titles and abstracts were screened. Of these, 189 remained for full-article screening; 52 articles were assessed for eligibility and 26 articles were excluded. A flow diagram is provided in Figure 1.

### Study Characteristics

Details of the cognitive and motor studies have been summarized in eTable 1 and eTable 2 in the Supplement, respectively. The 26 studies included 1455 children with POE and 2982 controls. There were 18 unique samples of children because some longitudinal studies reported on the same cohort.^29,30,31,32,33,34,35,36,37,38,39,40,41,42,43,44,45,46,47,48,49,50,51,52,53,54^ The studies were all from high-income countries and regions, including the United States (n = 11),^30,32,34,37,38,39,41,42,44,46,50^ Australia (n = 2),^31,33^ Europe (n = 4),^29,35,40,49^ and Israel (n = 1).^36^ Heroin was used in conjunction with polydrug ingestion in 10 studies,^35,36,44,45,49,50,51,52,53,54^ methadone in conjunction with polydrug ingestion in 13 studies,^29,30,31,32,33,37,38,39,41,42,43,47,48^ and unspecified opioids in conjunction with polydrug ingestion in 3 studies.^34,40,46^

Mean (SE) age at cognitive testing was 13 (1.58) months for the toddler group, 4.5 (0.38) years for the preschool group, and 13 (2.36) years for the school-aged group. Mean (SD) age at motor testing was 2 (0.45) years. Children were born between 1970 and 2004. Sixteen^30,32,34,36,37,38,39,41,42,43,44,46,47,50,52,53^ studies controlled for socioeconomic status, 19^29,31,32,33,35,36,37,39,40,41,43,45,47,48,49,50,51,53,54^ reported rates of NAS, and 21^29,30,31,33,35,36,37,38,40,41,43,44,45,46,47,48,50,51,52,53,54^ reported rates of foster or out-of-home care. The reported rates for NAS ranged from 53% to 93%. The incidence of NAS was not reported in 1 study, which was attributed to detoxification of the mothers by the third trimester.^49^ Rates of out-of-home care ranged from 20% to 72%. In 4 studies,^30,38,46,51^ all children with POE who were tested were in the care of their mother owing to the differences in recruitment methods, such as foster care being an exclusion criterion^46,51^ or having a subsample of mothers who were functional enough to retain custody of their children.^38^

### Cognitive Tests

For children aged 6 to 24 months, the Bayley Scales of Infant Development (BSID)^55^ was the most common cognitive test conducted (n = 8).^30,31,32,36,37,39,40,41^ Other tests included the Bayley Scales of Infant and Toddler Development–Second Edition (BSID-II)^56^ (n = 3),^33,34,35^ Bayley Scales of Infant and Toddler Development–Third Edition (BSID-III)^57^ (n = 1),^38^ and Griffiths Mental Development Scales^58^ (n = 1).^29^ For children 3 to 6 years, the most common test was the McCarthy Scales of Children’s Abilities^59^ (MSCA) (n = 5),^32,35,43,45,50^ followed by the Stanford-Binet Intelligence Scales^60^ (SB) (n = 4).^31,42,44,46^ Other tests included the Weschler Preschool and Primary Scale of Intelligence–III^61^ (n = 1),^49^ the Snijders-Oomen Nonverbal Intelligence tests^62^ (n = 1)^48^ and the Merrill-Palmer Scale of Mental Tests^63^ (n = 1).^47^ For children 7 to 18 years, the most common test was the Wechsler Intelligence Scale for Children^64^ (n = 2)^51,52^ or the Wechsler Intelligence Scale for Children–Revised^65^ (n = 1)^53^ (eTable 1 in the Supplement).

### Types of Motor Tests

The most common motor test used was the BSID^55^ (n = 7),^30,31,36,37,39,40,41^ followed by BSID-II^56^ (n = 2)^33,34^ and MSCA^59^ (n = 2).^32,54^ Other tests used were BSID-III^57^ (n = 1),^38^ Griffiths Mental Development Scales ^58^ (n = 1),^29^ and the Purdue Pegboard Test^66^ (n = 1)^44^ (eTable 2 in the Supplement).

### Cognitive Scores

**Figure 2.**
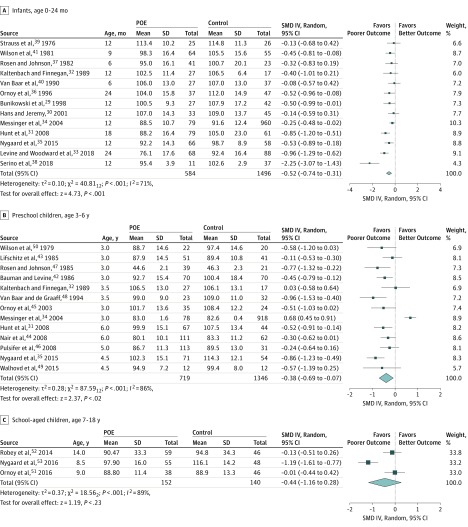
Cognitive Outcomes Among All Age Groups IV indicates inverse variance method; POE, prenatal opioid exposure; and SMD, standardized mean difference.

For infants aged 0 to 24 months, 13 studies pooling 584 children with POE and 1496 controls revealed a significant difference in neurocognitive development. Results for children with POE were lower (*d* = −0.52; 95% CI, −0.74 to −0.31; *P* < .001) than those of the controls (Figure 2A). Heterogeneity was significant at 71%. This incidence was partially accounted for by subgroup analysis including only studies that tested using the BSID (*I*^2^ = 35%) or included exposure to unspecified drugs (*I*^2^ = 0).

For preschool children aged 3 to 6 years, 13 studies pooling 719 children with POE and 1346 controls revealed a significant difference in neurocognitive development. Results for children with POE were lower (*d* = −0.38; 95% CI, −0.69 to −0.07; *P* < .02) than those of controls (Figure 2B). Heterogeneity was significant at 86%. This incidence was partially accounted for by subgroup analyses, including only studies that tested using the MSCA (*I*^2^ = 60%) and Stanford-Binet Intelligence Scales (*I^2^* = 0%) or children with exposure to methadone (*I*^2^ = 55%) and heroin (*I*^2^ = 0%).

For school-aged children 7 to 18 years, 3 studies pooling 152 children with POE and 140 controls showed that the difference in neurocognitive development was not significant (*d* = −0.44; 95% CI, −1.16 to 0.28; *P* = .23) (Figure 2C). Heterogeneity was significant at 89%. However, the number of studies was too small to perform subgroup analysis. All studies were considered high quality.

### Motor Outcomes

**Figure 3.**
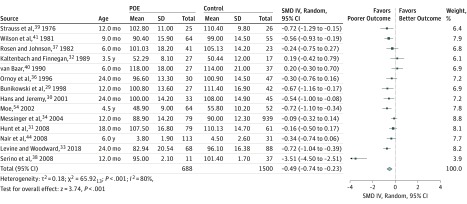
Motor Results in Children Aged 0 to 6 Years IV indicates inverse variance method; POE, prenatal opioid exposure; and SMD, standardized mean difference.

For all children 6 years or younger, 14 studies pooling 688 children with POE and 1500 controls revealed a significant difference in motor development. Results for children with POE were lower (*d* = −0.49; 95% CI, −0.74 to −0.23; *P* < .001) than those of the controls (Figure 3). Heterogeneity was significant at 80%. This incidence was partially accounted for by subgroup analyses, including only studies that tested using the BSID (*I*^2^ = 37%) or children exposed to heroin (*I*^2^ = 22).

### Factors Associated With Outcome Differences

Post hoc univariate metaregression analyzing the association between rates of NAS, rates of nonmaternal or foster care, and SMD between children with POE and controls was performed. No significant associations between rates of NAS and SMD were found for the age groups 6 to 24 months (B = 0.002; 95% CI, −0.013 to 0.016; *P* = .79) and 3 to 6 years (B = −0.00; 95% CI, −0.008 to 0.007; *P* = .96). Similarly, no significant associations between rates of nonmaternal care and SMD were found for the age groups 6 to 24 months (B = −0.001; 95% CI, −0.008 to 0.007; *P* = .82) and 3 to 6 years (B = −0.003; 95% CI, −0.014 to 0.008; *P* = .57).

### Evaluation of Bias

There was no evidence of publication bias in studies comparing cognitive scores of infants (SE, 1.05; 95% CI, −1.11 to 3.46; *P* = .13) and preschool children (SE, 2.15; 95% CI, −2.07 to 7.29; *P* = .19) using funnel plot inspection and the Egger test. Publication bias for adolescents was not assessed because there were inadequate numbers (n = 3) of included studies. There was evidence of publication bias in studies comparing motor scores of all children (SE, 1.71; 95% CI, 1.81-9.19; *P* = .004).

## Discussion

This systematic review and meta-analysis suggests that differences in neurocognitive testing associated with POE occur across a wide age range. The results agree with our hypothesis that POE has a negative association with cognitive and motor outcomes, these issues are apparent from as early as 6 months, and they persist during school age. To put this finding in perspective, an SMD of 0.38 to 0.52 corresponds to a moderate effect size,^25^ equivalent to 5.7 to 7.8 IQ points on a population level.^67,68^ Therefore, we expect that up to 6.3% of children with POE will have an IQ score 2 SDs below normal compared with 2.3% of children in a normally distributed population, suggesting that children with POE are 3 times more likely to have severe intellectual disability according to the *Diagnostic and Statistical Manual of Mental Disorders, 5th edition*^69^ criteria. This difference is significant for children with POE as they are already vulnerable given their tenuous living circumstances and increased risk of neglect and abuse,^11^ as well as their propensity to have behavioral and attention deficits,^70^ all of which contribute to poorer academic, social, and lifestyle outcomes.^30,34^

The results of our analysis of motor outcomes are similar to those of cognitive outcomes. Children with POE have poorer motor development compared with healthy controls. We found a difference of 0.49—a small to moderate effect size. Deficits in gross motor and fine motor function are associated with poorer executive function.^71^ Thus, our findings point to opioids being associated with overall neurodevelopment in infants and preschool children both directly and indirectly, as a child’s developmental trajectory is also influenced by his or her physical ability to experience and interact with the world.^72^

Poor neurodevelopmental outcomes in children with POE, even from an early age, is not novel information.^31,73^ However, our data appear to indicate that neurodevelopment did not improve after preschool and worsened by school age. The cause of this outcome is unclear. During preschool, children may receive considerably more individual attention than at a later time in education, with the reduced intervention possibly leading to worsening cognitive abilities. Regardless of the cause, this hampered neurodevelopment has serious implications. For example, a data linkage study by Oei et al^74^ demonstrated that performance on curriculum-based tests of Australian children with a history of NAS declined as the children aged and that, by high school, the results of the children with NAS were worse than those of children 2 years younger without NAS. However, there were a limited number of studies assessing children after school entry in the present meta-analysis, and this knowledge gap should be addressed in future studies.

There are considerable individual and societal consequences of poor neurocognitive performance. Neurocognitive performance is strongly correlated with future academic achievement.^75^ School underachievement is reported to lead to students dropping out after failing to meet examination requirements or finishing school with poorer qualifications.^76^ Such individuals receive lower wages and are more likely to be unemployed.^77^ Academic failure is also associated with youth delinquency^78^ as well as early initiation of alcohol and illicit substance use.^79^ High criminal rates and substance use further affect the country through the justice system and police expenditures, as well as public health care expenditures.^76^ Therefore, poor neurocognitive performance in childhood and adolescence may lead to financial problems for the individual owing to difficulties with employment and incur societal costs associated with youth delinquency and substance use. The consequences are likely to be passed on intergenerationally as the deficits associated with NAS are likely to influence parenting by NAS-affected adults.

Opioid substitution therapies limit fetal exposure to the lability of short-acting opioids, such as heroin, and stabilize the intrauterine environment.^80^ Opioid-dependent women who receive substitution therapy during their pregnancy are more stable psychologically and physically, receive more comprehensive antenatal care, and have better neonatal outcomes than women who are not receiving opioid substitution therapies.^81^ Our study is retrospective and observational; more studies need to be conducted before the current standard of care is changed.

In addition, the cause of these poor outcomes cannot be absolutely determined from the studies reviewed herein owing to the combination of inherited epigenetic changes,^70^ poor parental education, direct effect of opioids on brain volume,^17^ or the child’s home environment.^44^ Overall, there is substantial variability within each subgroup for neurocognitive outcomes as the children age, as evidenced by the widening 95% CIs, suggesting that children have the potential to overcome early discrepancies and environmental and other factors might possibly improve future outcomes. A meta-analysis examining the use of cognitive interventions for children with neurodevelopmental disorders^82^ has shown that improvement in neurocognitive functioning across all domains is possible, albeit to different degrees.

### Future Research

Our results suggest that conducting high-quality longitudinal cohort studies may be warranted to investigate the neurocognitive outcomes of children with POE until adolescence. In addition, studies using cohort and randomized trial designs should assess whether other factors (eg, foster care and parenting) contribute to the outcomes of children with POE. While we acknowledge that such studies will require substantial funding, personnel, and time, it is worthwhile since our results suggest that children with POE never reach the neurocognitive level of their peers, which increases their risk of poor school performance, unemployment,^76^ and even criminal activity.^78^ This outcome raises concern not only for the individual, but for his or her family, community, and society, considering the rapid rise of prescription opioid use and abuse around the world.

### Limitations

This meta-analysis has limitations. To include all available studies, we used hand-searching, which may have introduced citation bias. However, publication bias was not detected for cognitive outcomes. Although there was publication bias for motor outcomes, the trim-and-fill method, which is commonly used to correct for funnel plot asymmetry, is not recommended in this case owing to the high level of heterogeneity.^22^

Heterogeneity was significant for all analyses, although it was expected owing to the inclusion of multiple opioid types, various neurodevelopmental tests used, and clinical factors.^22^ Nevertheless, we attempted to explain the heterogeneity via subgroup analysis. Although heterogeneity was not completely accounted for, our results are based on a random-effects model, taking heterogeneity into consideration.

A key limitation is that we were unable to contact some authors for missing data, resulting in the exclusion of studies without means and SDs. We also had incomplete information on rates of NAS, rates of foster care, sex, parental educational levels, and substances used in pregnancy. In addition, the articles were limited to those published in English.

We performed post hoc metaregression on the association between rates of foster care, rates of NAS, and cognitive differences (SMD), but we did not find a significant association. One caveat is that post hoc analyses are not recommended because findings are not robust and are prone to inaccurate conclusions derived from observational patterns.^83^ Our intention was to generate hypotheses that could potentially explain our results and inform future studies.

## Conclusions

This systematic review and meta-analysis suggests that POE is negatively associated with neurocognitive and motor development. These differences begin from age 6 months and persist in adolescence. The exact cause and the association of these findings with clinical factors and environmental adversities are unclear but suggest that children with POE should be provided long-term support and intervention beyond infancy.

## Notes

Supplement. **eTable 1.** Descriptive Characteristics of Cognitive Outcomes **eTable 2.** Descriptive Characteristics of Motor Outcomes

## References

[1] Patrick SW, Dudley J, Martin PR, Prescription opioid epidemic and infant outcomes. Pediatrics. 2015;135(5):-. doi:10.1542/peds.2014-3299 25869370 PMC4411781

[2] Nørgaard M, Nielsson MS, Heide-Jørgensen U Birth and neonatal outcomes following opioid use in pregnancy: a Danish population-based study. Subst Abuse. 2015;9(suppl 2):5-11. 26512202 10.4137/SART.S23547 PMC4599593

[3] Patrick SW, Davis MM, Lehmann CU, Cooper WO Increasing incidence and geographic distribution of neonatal abstinence syndrome: United States 2009 to 2012. J Perinatol. 2015;35(8):650-655. doi:10.1038/jp.2015.36 25927272 PMC4520760

[4] Wu C-C, Hung C-J, Shen C-H, Prenatal buprenorphine exposure decreases neurogenesis in rats. Toxicol Lett. 2014;225(1):92-101. doi:10.1016/j.toxlet.2013.12.001 24321744

[5] Hu S, Sheng WS, Lokensgard JR, Peterson PK Morphine induces apoptosis of human microglia and neurons. Neuropharmacology. 2002;42(6):829-836. doi:10.1016/S0028-3908(02)00030-8 12015209

[6] McGinty JF, Ford DH Effects of prenatal methadone on rat brain catecholamines. Dev Neurosci. 1980;3(4-6):224-234. doi:10.1159/000112395 7460795

[7] Slotkin TA, Whitmore WL, Salvaggio M, Seidler FJ Perinatal methadone addiction affects brain synaptic development of biogenic amine systems in the rat. Life Sci. 1979;24(13):1223-1229. doi:10.1016/0024-3205(79)90059-6 571949

[8] Younger JW, Chu LF, D’Arcy NT, Trott KE, Jastrzab LE, Mackey SC Prescription opioid analgesics rapidly change the human brain. Pain. 2011;152(8):1803-1810. doi:10.1016/j.pain.2011.03.028 21531077 PMC3138838

[9] Parolin M, Simonelli A, Mapelli D, Sacco M, Cristofalo P Parental substance abuse as an early traumatic event: preliminary findings on neuropsychological and personality functioning in young drug addicts exposed to drugs early. Front Psychol. 2016;7:887. doi:10.3389/fpsyg.2016.00887 27378983 PMC4909766

[10] Kaltenbach K, O’Grady KE, Heil SH, Prenatal exposure to methadone or buprenorphine: early childhood developmental outcomes. Drug Alcohol Depend. 2018;185:40-49. doi:10.1016/j.drugalcdep.2017.11.030 29413437 PMC5906792

[11] Swift W, Copeland J, Hall W Characteristics of women with alcohol and other drug problems: findings of an Australian national survey. Addiction. 1996;91(8):1141-1150. doi:10.1046/j.1360-0443.1996.91811416.x 8828242

[12] Ellwood DA, Sutherland P, Kent C, O’Connor M Maternal narcotic addiction: pregnancy outcome in patients managed by a specialized drug-dependency antenatal clinic. Aust N Z J Obstet Gynaecol. 1987;27(2):92-98. doi:10.1111/j.1479-828X.1987.tb00952.x 3675451

[13] Abdel-Latif ME, Bajuk B, Lui K, Oei J; NSW on ACT Neonatal Intensive Care Units’ Study (NICUS) Group Short-term outcomes of infants of substance-using mothers admitted to neonatal intensive care units in New South Wales and the Australian Capital Territory. J Paediatr Child Health. 2007;43(3):127-133. doi:10.1111/j.1440-1754.2007.01031.x 17316185

[14] Taplin S, Mattick RP Mothers in methadone treatment and their involvement with the child protection system: a replication and extension study. Child Abuse Negl. 2013;37(8):500-510. doi:10.1016/j.chiabu.2013.01.003 23428166

[15] Griffiths SK, Campbell JP Placental structure, function and drug transfer. Contin Educ Anaesth Crit Care Pain. 2015;15(2):84-89. doi:10.1093/bjaceaccp/mku013

[16] Ross EJ, Graham DL, Money KM, Stanwood GD Developmental consequences of fetal exposure to drugs: what we know and what we still must learn. Neuropsychopharmacology. 2015;40(1):61-87. doi:10.1038/npp.2014.147 24938210 PMC4262892

[17] Walhovd KB, Moe V, Slinning K, Volumetric cerebral characteristics of children exposed to opiates and other substances in utero. Neuroimage. 2007;36(4):1331-1344. doi:10.1016/j.neuroimage.2007.03.070 17513131 PMC2039875

[18] Nygaard E, Slinning K, Moe V, Due-Tønnessen P, Fjell A, Walhovd KB Neuroanatomical characteristics of youths with prenatal opioid and poly-drug exposure. Neurotoxicol Teratol. 2018;68:13-26. doi:10.1016/j.ntt.2018.04.004 29679636

[19] McDaniel MA Big-brained people are smarter: a meta-analysis of the relationship between in vivo brain volume and intelligence. Intelligence. 2005;33(4):337-346. doi:10.1016/j.intell.2004.11.005

[20] Stroup DF, Berlin JA, Morton SC, ; Meta-analysis of Observational Studies in Epidemiology (MOOSE) Group Meta-analysis of observational studies in epidemiology: a proposal for reporting. JAMA. 2000;283(15):2008-2012. doi:10.1001/jama.283.15.2008 10789670

[21] Liberati A, Altman DG, Tetzlaff J, The PRISMA statement for reporting systematic reviews and meta-analyses of studies that evaluate healthcare interventions: explanation and elaboration. BMJ. 2009;339:b2700. doi:10.1136/bmj.b2700 19622552 PMC2714672

[22] Higgins J, Green S, eds. *Cochrane Handbook for Systematic Reviews of Interventions* 5.1.0 ed. London, England: The Cochrane Collaboration; 2011.

[23] Wallace BC, Schmid CH, Lau J, Trikalinos TA Meta-Analyst: software for meta-analysis of binary, continuous and diagnostic data. BMC Med Res Methodol. 2009;9(1):80. doi:10.1186/1471-2288-9-80 19961608 PMC2795760

[24] Riley RD, Higgins JPT, Deeks JJ Interpretation of random effects meta-analyses. BMJ. 2011;342:d549. doi:10.1136/bmj.d549 21310794

[25] Cochrane Handbook for Systematic Reviews of Interventions 12.6.2 Re-expressing SMDs using rules of thumb for effect sizes. https://handbook-5-1.cochrane.org/chapter_12/12_6_2_re_expressing_smds_using_rules_of_thumb_for_effect_sizes.htm. Accessed August 5, 2018.

[26] Sterne JAC, Egger M, Smith GD Systematic reviews in health care: investigating and dealing with publication and other biases in meta-analysis. BMJ. 2001;323(7304):101-105. doi:10.1136/bmj.323.7304.101 11451790 PMC1120714

[27] Sedgwick P Meta-analyses: what is heterogeneity? BMJ. 2015;350:h1435. doi:10.1136/bmj.h1435 25778910

[28] Wells G, Shea B, O’Connell D, The Newcastle-Ottawa Scale (NOS) for assessing the quality of nonrandomised studies in meta-analyses. http://www.ohri.ca/programs/clinical_epidemiology/oxford.asp. Accessed August 30, 2018.

[29] Bunikowski R, Grimmer I, Heiser A, Metze B, Schäfer A, Obladen M Neurodevelopmental outcome after prenatal exposure to opiates. Eur J Pediatr. 1998;157(9):724-730. doi:10.1007/s004310050923 9776530

[30] Hans SL, Jeremy RJ Postneonatal mental and motor development of infants exposed in utero to opioid drugs. Infant Ment Health J. 2001;22(3):300-315. doi:10.1002/imhj.1003

[31] Hunt RW, Tzioumi D, Collins E, Jeffery HE Adverse neurodevelopmental outcome of infants exposed to opiate in-utero. Early Hum Dev. 2008;84(1):29-35. doi:10.1016/j.earlhumdev.2007.01.013 17728081

[32] Kaltenbach K, Finnegan LP Children exposed to methadone in utero: assessment of developmental and cognitive ability. Ann N Y Acad Sci. 1989;562(1):360-362. doi:10.1111/j.1749-6632.1989.tb21039.x

[33] Levine TA, Woodward LJ Early inhibitory control and working memory abilities of children prenatally exposed to methadone. Early Hum Dev. 2018;116:68-75. doi:10.1016/j.earlhumdev.2017.11.010 29195088

[34] Messinger DS, Bauer CR, Das A, The maternal lifestyle study: cognitive, motor, and behavioral outcomes of cocaine-exposed and opiate-exposed infants through three years of age. Pediatrics. 2004;113(6):1677-1685. doi:10.1542/peds.113.6.1677 15173491

[35] Nygaard E, Moe V, Slinning K, Walhovd KB Longitudinal cognitive development of children born to mothers with opioid and polysubstance use. Pediatr Res. 2015;78(3):330-335. doi:10.1038/pr.2015.95 25978800 PMC4539602

[36] Ornoy A, Michailevskaya V, Lukashov I, Bar-Hamburger R, Harel S The developmental outcome of children born to heroin-dependent mothers, raised at home or adopted. Child Abuse Negl. 1996;20(5):385-396. doi:10.1016/0145-2134(96)00014-2 8735375

[37] Rosen TS, Johnson HL Children of methadone-maintained mothers: follow-up to 18 months of age. J Pediatr. 1982;101(2):192-196. doi:10.1016/S0022-3476(82)80115-7 6178811

[38] Serino Ma D, Peterson Md BS, Rosen Md TS Psychological functioning of women taking illicit drugs during pregnancy and the growth and development of their offspring in early childhood. J Dual Diagn. 2018;14(3):158-170. doi:10.1080/15504263.2018.1468946 29694295 PMC6202263

[39] Strauss ME, Starr RH, Ostrea EM, Chavez CJ, Stryker JC Behavioural concomitants of prenatal addiction to narcotics. J Pediatr. 1976;89(5):842-846. doi:10.1016/S0022-3476(76)80822-0 978336

[40] van Baar A Development of infants of drug dependent mothers. J Child Psychol Psychiatry. 1990;31(6):911-920. doi:10.1111/j.1469-7610.1990.tb00833.x 2246341

[41] Wilson GS, Desmond MM, Wait RB Follow-up of methadone-treated and untreated narcotic-dependent women and their infants: health, developmental, and social implications. J Pediatr. 1981;98(5):716-722. doi:10.1016/S0022-3476(81)80830-X 6164775

[42] Bauman PS, Levine SA The development of children of drug addicts. Int J Addict. 1986;21(8):849-863. doi:10.3109/10826088609027399 3771015

[43] Lifschitz MH, Wilson GS, Smith EO, Desmond MM Factors affecting head growth and intellectual function in children of drug addicts. Pediatrics. 1985;75(2):269-274. 3969327

[44] Nair P, Black MM, Ackerman JP, Schuler ME, Keane VA Children’s cognitive-behavioral functioning at age 6 and 7: prenatal drug exposure and caregiving environment. Ambul Pediatr. 2008;8(3):154-162. doi:10.1016/j.ambp.2008.02.002 18501861 PMC2766549

[45] Ornoy A The impact of intrauterine exposure versus postnatal environment in neurodevelopmental toxicity: long-term neurobehavioral studies in children at risk for developmental disorders. Toxicol Lett. 2003;140-141:171-181. doi:10.1016/S0378-4274(02)00505-2 12676464

[46] Pulsifer MB, Butz AM, O’Reilly Foran M, Belcher HME Prenatal drug exposure: effects on cognitive functioning at 5 years of age. Clin Pediatr (Phila). 2008;47(1):58-65. doi:10.1177/0009922807305872 17766581 PMC2269702

[47] Rosen TS, Johnson HL Long-term effects of prenatal methadone maintenance. NIDA Res Monogr. 1985;59:73-83. 3929135

[48] van Baar A, de Graaff BM Cognitive development at preschool-age of infants of drug-dependent mothers. Dev Med Child Neurol. 1994;36(12):1063-1075. doi:10.1111/j.1469-8749.1994.tb11809.x 7958521

[49] Walhovd KB, Bjørnebekk A, Haabrekke K, Child neuroanatomical, neurocognitive, and visual acuity outcomes with maternal opioid and polysubstance detoxification. Pediatr Neurol. 2015;52(3):326-32.e1, 3. doi:10.1016/j.pediatrneurol.2014.11.008 25595574

[50] Wilson GS, McCreary R, Kean J, Baxter JC The development of preschool children of heroin-addicted mothers: a controlled study. Pediatrics. 1979;63(1):135-141. doi:10.1016/S0022-3476(79)80107-9 86983

[51] Ornoy A, Finkel-Pekarsky V, Peles E, Adelson M, Schreiber S, Ebstein PR ADHD risk alleles associated with opiate addiction: study of addicted parents and their children. Pediatr Res. 2016;80(2):228-236. doi:10.1038/pr.2016.78 27064247

[52] Robey A, Buckingham-Howes S, Salmeron BJ, Black MM, Riggins T Relations among prospective memory, cognitive abilities, and brain structure in adolescents who vary in prenatal drug exposure. J Exp Child Psychol. 2014;127:144-162. doi:10.1016/j.jecp.2014.01.008 24630759 PMC4133286

[53] Nygaard E, Slinning K, Moe V, Walhovd KB Behavior and attention problems in eight-year-old children with prenatal opiate and poly-substance exposure: a longitudinal study. PLoS One. 2016;11(6):e0158054. doi:10.1371/journal.pone.0158054 27336798 PMC4918960

[54] Moe V Foster-placed and adopted children exposed in utero to opiates and other substances: prediction and outcome at four and a half years. J Dev Behav Pediatr. 2002;23(5):330-339. doi:10.1097/00004703-200210000-00006 12394521

[55] Bayley N Bayley Scales of Infant Development. San Antonio, TX: Psychological Corp; 1969.

[56] Bayley N Bayley Scales of Infant Development. 2nd ed San Antonio, TX: Psychological Corp; 1993.

[57] Bayley N Bayley Scales of Infant Development. 3rd ed San Antonio, TX: Harcourt Assessment; 2006.

[58] Griffiths R The Abilities of Young Children. High Wydcombe, UK: The Test Agency; 1976.

[59] McCarthy D Manual for the McCarthy Scales of Children’s Abilities. New York, NY: Psychological Corp; 1972.

[60] Gale HR Stanford-Binet Intelligence Scales. 5th ed Itasca, IL: Riverside Publishing; 2003.

[61] Weschler D The Wechsler Preschool and Primary Scale of Intelligence. 3rd ed San Antonio, TX: The Psychological Corp; 1989.

[62] Snijders JT, Snijders-Oomen N *Snijders-Oomen Non-verbal Intelligence Scale, SON 2.5-7* Groningen, the Netherlands: Wolters-Noordhoff; 1976.

[63] Stutsman R Merrill-Palmer Scale of Mental Tests. Los Angeles, CA: Western Psychological Services; 1948.

[64] Weschler D Wechsler Intelligence Scale for Children. San Antonio, TX: Psychological Corp; 1949.

[65] Weschler D Wechsler Intelligence Scale For Children-Revised. New York, NY: Psychological Corp; 1974.

[66] Tiffin J, Asher EJ The Purdue pegboard; norms and studies of reliability and validity. J Appl Psychol. 1948;32(3):234-247. doi:10.1037/h0061266 18867059

[67] Brydges CR, Landes JK, Reid CL, Campbell C, French N, Anderson M Cognitive outcomes in children and adolescents born very preterm: a meta-analysis. Dev Med Child Neurol. 2018;60(5):452-468. doi:10.1111/dmcn.13685 29453812

[68] Twilhaar ES, Wade RM, de Kieviet JF, van Goudoever JB, van Elburg RM, Oosterlaan J Cognitive outcomes of children born extremely or very preterm since the 1990s and associated risk factors: a meta-analysis and meta-regression. JAMA Pediatr. 2018;172(4):361-367. doi:10.1001/jamapediatrics.2017.5323 29459939 PMC5875339

[69] American Psychiatric Association. Diagnostic and Statistical Manual of Mental Disorders. 5th ed. Arlington, VA: American Psychiatric Association; 2013.

[70] Maguire DJ, Taylor S, Armstrong K, Long-term outcomes of infants with neonatal abstinence syndrome. Neonatal Netw. 2016;35(5):277-286. doi:10.1891/0730-0832.35.5.277 27636691

[71] Gonzalez SL, Reeb-Sutherland BC, Nelson EL Quantifying motor experience in the infant brain: EEG power, coherence, and mu desynchronization. Front Psychol. 2016;7(216):216. 26925022 10.3389/fpsyg.2016.00216 PMC4757680

[72] Libertus K, Hauf P Motor skills and their foundational role for perceptual, social, and cognitive development. Front Psychol. 2017;8:301. doi:10.3389/fpsyg.2017.00301 28321199 PMC5337521

[73] Baldacchino A, Arbuckle K, Petrie DJ, McCowan C Neurobehavioral consequences of chronic intrauterine opioid exposure in infants and preschool children: a systematic review and meta-analysis. BMC Psychiatry. 2014;14(1):104. doi:10.1186/1471-244X-14-104 24708875 PMC4021271

[74] Oei JL, Melhuish E, Uebel H, Neonatal abstinence syndrome and high school performance. Pediatrics. 2017;139(2):e20162651. doi:10.1542/peds.2016-2651 28093465

[75] Yen C-J, Konold TR, McDermott PA Does learning behavior augment cognitive ability as an indicator of academic achievement? J Sch Psychol. 2004;42(2):157-169. doi:10.1016/j.jsp.2003.12.001

[76] Organisation for Economic Cooperation and Development 2010 Overcoming school failure: policies that work. http://www.oecd.org/education/school/45171670.pdf. Accessed August 5, 2018.

[77] Jimerson SR On the failure of failure: examining the association between early grade retention and education and employment outcomes during late adolescence. J Sch Psychol. 1999;37(3):243-272. doi:10.1016/S0022-4405(99)00005-9

[78] Christle C, Jolivette K, Nelson C Breaking the school to prison pipeline: identifying school risk and protective factors for youth delinquency. Exceptionality. 2005;13(2):69-88. doi:10.1207/s15327035ex1302_2

[79] Trenz RC, Harrell P, Scherer M, Mancha BE, Latimer WW A model of school problems, academic failure, alcohol initiation, and the relationship to adult heroin injection. Subst Use Misuse. 2012;47(10):1159-1171. doi:10.3109/10826084.2012.686142 22621313 PMC6280666

[80] Winklbaur B, Kopf N, Ebner N, Jung E, Thau K, Fischer G Treating pregnant women dependent on opioids is not the same as treating pregnancy and opioid dependence: a knowledge synthesis for better treatment for women and neonates. Addiction. 2008;103(9):1429-1440. doi:10.1111/j.1360-0443.2008.02283.x 18783498

[81] Hudak ML, Tan RC; Committee on Drugs; Committee on Fetus and Newborn; American Academy of Pediatrics Neonatal drug withdrawal. Pediatrics. 2012;129(2):e540-e560. doi:10.1542/peds.2011-3212 22291123

[82] Robinson KE, Kaizar E, Catroppa C, Godfrey C, Yeates KO Systematic review and meta-analysis of cognitive interventions for children with central nervous system disorders and neurodevelopmental disorders. J Pediatr Psychol. 2014;39(8):846-865. doi:10.1093/jpepsy/jsu031 24864276

[83] Thompson SG, Higgins JP How should meta-regression analyses be undertaken and interpreted? Stat Med. 2002;21(11):1559-1573. doi:10.1002/sim.1187 12111920

