# Supplementary material for: Cognitive and Motor Outcomes of Children With Prenatal Opioid Exposure: A Systematic Review and Meta-analysis
Source: JAMA Netw Open. 2019 Jul 12;2(7):e197025. doi: 10.1001/jamanetworkopen.2019.7025 (PMC6628595; doi:10.1001/jamanetworkopen.2019.7025)
Supplement: Supplement. — eTable 1. Descriptive Characteristics of Cognitive Outcomes eTable 2. Descriptive Characteristics of Motor Outcomes [file jamanetwopen-2-e197025-s001.pdf]

Supplementary Online Content

Yeoh SL, Eastwood J, Wright IM, et al. Cognitive and motor outcomes of children with prenatal opioid exposure: a systematic review and meta-analysis. *JAMA Netw Open*. 2019;2(7):e197025. doi:10.1001/jamanetworkopen.2019.7025

eTable 1. Descriptive Characteristics of Cognitive Outcomes

eTable 2. Descriptive Characteristics of Motor Outcomes

This supplementary material has been provided by the authors to give readers additional information about their work.

© 2019 Yeoh SL et al. *JAMA Network Open.*

eTable 1. Descriptive characteristics of cognitive outcomes.

			POE children					Control children			
**Study**	**Place of birth**	**Test**	**IQ, M (SD)**	**n**	**Main opioid used**	**NAS (%)**	**Out-of-home (%)**	**IQ, M(SD)**	**n**	**Age at assessment**	**Control for SES**
Bunikowski 1998[29]	Berlin	Griffiths	100.5 (9.3)	27	Methadone	91	65	107.9 (17.2)	42	12 months	No
Hans 2001[30]	USA	BSID	107 (14.3)	33	Methadone	NR	0	109 (13.7)	45	12 months	Yes
Hunt 2008[31]	Australia	BSID	88.2 (16.4)	79	Methadone	56	16	105.02 (23.0)	61	18 months	No
		SB	99.9 (15.1)	67				107.5 (13.4)	44	3 years	
Kaltenbach 1989[32]	USA	BSID	102.5 (11.38)	27	Methadone	92	NR	106.53 (6.41)	17	12 months	Yes
		MSCA	106.51 (12.96)	27				106.05 (13.10)	17	3.5 years	
Levine 2017[33]	New Zealand	BSID-II	76.09 (17.63)	68	Methadone	88	21	92.44 (16.39)	88	24 months	No
Messinger 2004[34]	USA	BSID-II	88.5 (10.7)	79	Unspecified	NR	34.7	91.6 (12.4)	960	12 months	Yes
			83.0 (1.6)	78				82.6 (0.4)	918	3 years	
Nygaard 2015[35]	Oslo	BSID-II	92.2 (14.3)	66	Heroin	80	72	98.7 (8.9)	58	12 months	No
		MSCA	102.3 (15.1)	71				114.3 (12.1)	54	3 years	
Ornoy 1996[36]	Jerusalem	BSID	104 (15.8)	37	Heroin	74	62	112 (14.9)	47	24 months	Yes
Rosen 1982[37]	USA	BSID	95 (16.1)	41	Methadone	75	NR	100.69 (20.1)	23	6 months	Yes
Serino 2018[38]	USA	BSID-III	95.40 (3.90)	11	Methadone	NR	0	102.60 (2.90)	37	12 months	Yes
Strauss 1976[39]	USA	BSID	113.4 (10.2)	25	Methadone	65	NR	114.8 (11.3)	26	12 months	Yes
van Baar 1990[40]	Amsterdam	BSID	106 (13)	27	Unspecified	80	20	107 (13))	37	6 months	No
Wilson 1981[41]	USA	BSID	98.3 (16.4)	64	Methadone	93	33	105.5 (15.6)	55	9 months	Yes
Bauman 1986[42]	USA	SB	92.71 (15.36)	70	Methadone	NR	NR	100.41 (18.36)	70	6 years	Yes
Lifschitz 1985[43]	USA	MSCA	87.9 (14.5)	51	Methadone	78	65	89.4 (10.8)	41	6 years	Yes

© 2019 Yeoh SL et al. *JAMA Network Open.*

Nair 2008[44]	USA	SB	80.1 (10.1)	111	Heroin	NR	44.2	83.3 (11.2)	62	6 years	Yes
Ornoy 2003[45]	Jerusalem	MSCA	101.7 (13.6)	35	Heroin	74	62	108.4 (12.2)	24	6 years	No
**Study**	**Place of birth**	**Test**	**IQ, M (SD)**	**n**	**Main opioid used**	**NAS (%)**	**Out-of-home (%)**	**IQ, M(SD)**	**n**	**Age at assessment**	**Control for SES**
Pulsifier 2008[46]	USA	SB	86.7 (11.3)	113	Unspecified	NR	0	89.5 (13.0)	31	5 years	Yes
Rosen 1985[47]	USA	MPSMT	44.6 (2.1)	39	Methadone	75	NR	46.3 (2.3)	21	3 years	Yes
van Baar 1994[48]	Amsterdam	SON	99 (9)	23	Methadone	83	22	109 (11)	32	3.5 years	No
Walhovd 2015[49]	Oslo	WPPSI-III	94.9 (7.2)	12	Heroin	0	25	99.4 (8.0)	12	4.5 years	No
Wilson 1979[50]	USA	MSCA	88.71 (14.62)	22	Heroin	55	50	97.42 (14.62)	20	6 years	Yes
Nygaard 2016[53]	Oslo	WISC-R	97.9 (16)	55	Heroin	80	72	116.1(14.2)	48	8.5 years	Yes
Robey 2014[52]	USA	WISC	90.47 (33.3)	59	Heroin	NR	32	94.76 (34.29)	46	14 years	Yes
Ornoy 2016[51]	Jerusalem	WISC	88.8 (11.4)	38	Heroin	53	0	88.9 (13.3)	46	16.5 years	No

Abbreviations. BSID= Bayley Scales of Infant and Toddler Development, BSID-II= Bayley Scales of Infant and Toddler Development- Second Edition, BSID-III= Bayley Scales of Infant and Toddler Development-Third Edition, GMDS= Griffiths Mental Development Scales, MSCA= McCarthy Scales of Children's Abilities, SB= Stanford Binet Intelligence Scale, WISC = Wechsler Intelligence Scale for Children, WPPSI-III = Wechsler Preschool and Primary Scale of Intelligence- Third Edition, SON= Snijders-Oomen nonverbal intelligence test, MPSMT= Merill-Palmer Scale of Intelligence Test.

© 2019 Yeoh SL et al. *JAMA Network Open.*

eTable 2. Descriptive characteristics of motor outcomes.

		POE children					Control children			
**Study**	**Test**	**Motor, M (SD)**	**n**	**Main opioid used**	**NAS (%)**	**Out-of-home (%)**	**Motor, M(SD)**	**n**	**Age at assessment**	**Control for SES**
Bunikowski 1998[29]	Griffiths Locomoto r	100.8 (13.6)	27	Methadone	91	65	111.4 (16.9)	42	12 months	No
Hans 2001[30]	BSID	100 (14.2)	33	Methadone	NR	0	108 (14.9)	45	24 months	Yes
Levine 2017[33]	BSID-II	82.94 (20.54)	68	Methadone	88	21	96.10 (16.38)	88	24 months	No
Messinger 2004[34]	BSID-II	88.9 (14.2)	79	Unspecified	NR	34.7	90.0 (12.3)	939	12 months	Yes
Ornoy 1996[36]	BSID	96.6 (13.3)	30	Heroin	74	62	100.9 (14.5)	47	24 months	Yes
Rosen 1982[37]	BSID	101.03 (18.2)	41	Methadone	75	NR	105.13 (14.2)	23	6 months	Yes
Serino 2018[38]	BSID-III	95 (2.1)	11	Methadone	NR	0	101.4 (1.7)	37	12 months	Yes
Strauss 1976[39]	BSID	102.8 (11)	25	Methadone	65	NR	110.4 (9.8)	26	12 months	Yes
van Baar 1990[40]	BSID	118 (18)	27	Unspecified	80	20	114 (21)	37	6 months	No
Wilson 1981[41]	BSID	90.4 (15.9)	64	Methadone	93	33	99 (14.5)	55	9 months	Yes
Hunt 2008[31]	BSID	107.5 (16.8)	79	Methadone	56	16	110.13 (14.7)	61	18 months	No
Kaltenbach 1989[32]	MSCA-motor	52.29 (8.1)	27	Methadone	92	NR	50.44 (12)	17	4 years	No
Nair 2008[44]	Purdue Pegboard	3.8 (1.9)	113	Heroin	NR	44.2	4.5 (2.6)	31	6 years	Yes
Moe 2002[54]	MSCA-motor	48.9 (9.0)	64	Heroin	79.7	100	55.8 (10.2)	52	4.5 years	No

Abbreviations. BSID= Bayley Scales of Infant and Toddler Development, BSID-II= Bayley Scales of Infant and Toddler Development- Second Edition, BSID-III= Bayley Scales of Infant and Toddler Development-Third Edition, GMDS= Griffiths Mental Development Scales, MSCA= McCarthy Scales of Children's Abilities, Purdue Pegboard= Purdue Pegboard Test.

© 2019 Yeoh SL et al. *JAMA Network Open.*
